# Omega-3 Index and Sudden Cardiac Death

**DOI:** 10.3390/nu2030375

**Published:** 2010-03-23

**Authors:** Clemens von Schacky

**Affiliations:** 1 Preventive Cardiology, Medizinische Klinik and Poliklinik Innenstadt, Ludwig Maximilians University Munich, Ziemssenstr. 1, 80336 Munich, Germany; Email: Clemens.vonschacky@med.uni-muenchen.de; 2 Omegametrix, Am Klopferspitz 19, 82152 Martinsried, Germany; Email: c.vonschacky@omegametrix.eu

**Keywords:** sudden cardiac death, omega-3 Index, omega-3 fatty acids, eicosapentaenoic acid, docosahexaenoic acid, myocardial infarction

## Abstract

Sudden cardiac death (SCD) is an unresolved health issue, and responsible for 15% of all deaths in Western countries. Epidemiologic evidence, as well as evidence from clinical trials, indicates that increasing intake and high levels of eicosapentaenoic acid (EPA) and docosahexaenoic acid (DHA) protect from SCD and other major adverse cardiac events. Levels of EPA+DHA are best assessed by the Omega-3 Index, representing the red cell fatty acid content of EPA+DHA. Work is in progress that will further define the value of the Omega-3 Index as a risk factor for SCD, other cardiac events, and as target for treatment with EPA+DHA.

## 1. Introduction

Sudden cardiac death (SCD) is defined as a natural death occurring within one hour from onset of symptoms [1]. In the majority of cases, an acute cardiac event, like a myocardial infarction, precipitates ventricular tachycardia, which then degenerates into ventricular fibrillation with circulatory arrest. A 24 hour definition of SCD, previously frequently used, increases the fraction of all natural deaths falling into the “sudden” category, but reduces the proportion of all sudden natural deaths that are due to cardiac causes [1]. In Germany, similar to in other Western countries, SCD causes some 15 % of all deaths, and therefore is a major health problem [1]. This figure has not changed drastically over the years in spite of efforts to reduce the burden of SCD [1]. Efforts at preventing SCD have focused on identifying risk factors, but so far none have been found to be of proven predictive value in the general population [2]. However, the vast majority of SCDs terminate lives in the general population, *i.e.,* lives of “healthy” persons. Taken together, SCD abruptly ends productive lives.

Attempts to improve treatment of SCD have explored Automated External Defibrillators. They are installed in public places in the hope of reducing the burden of SCD. A positive effect of providing an automated external defibrillator to households with a member at high risk for SCD could not be proven [3]. To date, convincing evidence that treatment of SCD can be improved, is lacking. 

Attempts to improve prevention of SCD have focused on implantable automated defibrillators (ICDs) [4]. Currently, ICDs are only implanted in a small minority of patients fulfilling the MADIT-2 criteria [4]. Several randomized trials trying to broaden the patient population suitable for implantation of an ICD have failed [e.g., reference 5]. It should be noted, however, that guideline-conforming treatment of cardiac diseases, e.g., with beta-blockers or with inhibitors of the renin-angiotensin system, also reduces the incidence of SCD, although this evidence was provided by retrospective analyses of randomized controlled trials with other primary endpoints [6]. Taken together, the incidence of SCD has not measurably decreased in the last ten years [1].

Clearly, other approaches need to be explored to prevent SCD. The most promising approach to date is the use of the two marine omega-3 fatty acids: eicosapentaenoic acid (EPA) and docosahexaenoic acid (DHA). This article summarizes the present knowledge in this field with special reference to the Omega-3 Index.

## 2. Omega-3 Index

The Omega-3 Index was defined in 2004 as the percentage of EPA+DHA in red cell lipids [7]. For determination of the Omega-3 Index, a small volume of EDTA blood is required. The definition of the Omega-3 Index includes a highly standardized analytical laboratory methodology [7]. This methodology has been installed in three laboratories in the world (US, Germany, Korea), and was successfully subjected to an inter-laboratory comparison, *i.e.,* proficiency testing [8]. A high analytical reproducibility with a low coefficient of variation (4%) was found, a prerequisite to acknowledge the low biological variability of the Omega-3 Index [9,10]. The low biological variability may be due to the fact that the half-life of RBC EPA+DHA is 4–6 times longer than that of serum EPA+DHA [9]. Also, RBC fatty acid composition is less influenced by day-to-day variations and by dyslipidemias than are plasma fatty acids, which contributes to the fact that the Omega-3 Index is unaffected by the fasting or fed state [9,10]. In contrast to other assessments of levels of omega-3 fatty acids, like in plasma, the Omega-3 Index correlates with human cardiac ventricular or atrial tissue levels during steady intake of EPA and DHA, as well as after an increase in intake [11,12,13]. Therefore, the Omega-3 Index can be considered a long-term parameter reflecting a persons´ status in EPA and DHA. Short-term intake is better reflected by measurements of plasma fatty acid compartments [14,15]. A pre-analytical advantage is that samples are stable for seven days at room temperature, and can be shipped by regular mail, if taken into EDTA-coated tubes [9]. Taken together, from a methodological point of view, determining the Omega-3 Index has distinct advantages over determining levels of EPA+DHA in other fatty acid compartments. 

The level of the Omega-3 Index is influenced by intake of EPA and DHA: every 4 g of EPA and DHA ingested per month increased the Omega-3 Index by 0.24 % [16]. The Omega-3 Index is also influenced by age (+0.50% per decade), diabetes (−1.13% if present), body mass index (−0.30% per three units), gender, physical activity, and a number of other factors, like social status or alcohol intake [16,17,18,19]. Only a part of the population converts some alpha-linolenic acid to EPA, whereas another part is unable to perform this metabolic step [20]. Conversion of EPA to DHA is negligible [20]. The differences in conversion mentioned and the gender differences argue for a genetic influence on the Omega-3 Index. Other factors, yet to be defined, may also play a role.

Taken together, the Omega-3 Index can be considered a biomarker for a persons´ status in EPA and DHA. As to be expected for a biomarker, levels of the Omega-3 Index have a normal (Gaussian) distribution in all populations studied [21,22].

## 3. Epidemiology of Sudden Cardiac Death

The incidence of SCD in Western countries is 20-fold the incidence of SCD in Japan [1,23]. In the US, in Kansas City, an average Omega-3 Index of 4.9%, in Germany of 5.6%, in Japan of approx. 8.5% and in Korea of 11%, was found in general populations [8,16,17,21,22]. Within a population, in Seattle, the risk for SCD with a low percentage of EPA+DHA in red cell membranes was ten-fold the risk for SCD with a high percentage of EPA+DHA [24]. These findings were mirrored in the Physicians Health Study, where whole blood was analyzed, and a ten-fold difference in risk for SCD found [25]. Data on fish consumption and SCD are in line with these observations, but less pronounced: an example is the Zutphen study, where long-term fatty-fish consumption was associated with lower risk of SCD [HR: 0.46 (0.27–0.78), p for trend 0.18] [26]. 

The epidemiologic data on SCD are supported by epidemiologic data on fatal myocardial infarction, a frequent cause of SCD. Using the MONICA criteria, up to 60-fold differences in incidence of fatal myocardial infarction were found when comparing Japanese with some Western populations [27]. Of note, studies comparing Japanese living in Japan with Japanese living in Western countries found higher incidences of SCD and fatal myocardial infarction in Japanese living in Western countries [21].

Although the frequency of conventional risk factors in Japanese living in Japan is similar to or even higher than for Japanese living in Western countries, atherosclerosis is more pronounced in Japanese living in Western countries [28]. Several lines of evidence indicate that this, and the lower incidence of cardiac deaths in Japanese living in Japan as compared to Japanese living in Western countries, does not seem to be due to genetic differences, but rather to environmental factors, such as diet [28]. This is reflected in part in the higher levels of EPA+DHA in Japanese living in Japan than in Japanese living in Western countries [21].

There is no evidence that increased fish consumption is related to a compensatory increase in mortality from other causes, like cancer [29]. 

## 4. Mechanisms of Action and Animal Models

EPA+DHA are incorporated into myocardial cells [11,12,13], which is considered to have profound effects on trafficking of channels through subcellular compartments and in lipid rafts [30]. *In vitro*, concentration-dependent inhibition of sodium channels, and of various potassium channels has been demonstrated [30]. Less data have been generated for calcium channels [30]. More complex actions and interactions between omega-3 fatty acids and ion channels have been demonstrated, and are currently actively being investigated [30]. In an *ex vivo *model, heart cells of patients with heart failure were superfused with physiological concentrations of EPA+DHA [31]. Triggered arrhythmias were inhibited, due to a decrease in intracellular calcium and a reduced response to noradrenalin [31].

Animal models have also been investigated [30]. The advantage of animal studies is that they allow studying variables not easily accessible in humans. On the other hand, extrapolating the findings to humans can be difficult. An example is the fact that EPA+DHA usually lower heart rate in humans, whereas in experimental animals they do not [30], implying fundamental differences in electrophysiology. In general, chronic feeding experiments demonstrated an anti-arrhythmic effect of EPA+DHA, while some exceptions have been reported [30]. Short-term anti-arrhythmic effects were also observed in infusion experiments [30].

## 5. Studies in Humans on Surrogate and Intermediate Parameters

In epidemiologic studies, a high resting heart rate is associated with decreased survival and increased SCD [1]. In 2005, a meta-analysis of randomized controlled trials found resting heart rate to be reduced by 2.5 beats per minute, if >69 beats per minute at baseline [32]. The results of more recent studies were in line [33]. Similarly, a low heart rate variability is also associated with decreased survival and increased SCD [1]. Although a formal meta-analysis remains to be performed, most, but not all, intervention studies demonstrated an increase in heart rate variability after increased ingestion of EPA+DHA [33].

Inducibility of ventricular tachycardia, a parameter assessed in a cardiac catheter laboratory, was found to be suppressed in controlled studies in humans after either six weeks oral EPA+DHA or in acute infusion studies [34,35].

Three randomized controlled intervention studies were conducted in carriers of implanted cardioverters/defibrillators. No difference in the primary endpoint of these studies was seen: any action of the device, shock or anti-tachycardia pacing [36]. Total mortality in the fish oil groups was 25, and in the placebo groups 36 patients, a non-significant risk reduction of 30% (RR 0.70, 95% CI 0.42–1.15, *p* = 0.15) [36]. More recently, it has been demonstrated that in patients with a high omega-3 index, ICDs shocked less frequently than in patients with a low omega-3 index [37]. However, in patients with low levels of EPA+DHA in their red cells, ventricular tachycardias were less frequently terminated by antitachycardia pacing than in patients with high levels [38]. In patients with an ICD; life expectancy after a shock is much shorter than after anti-tachycardia pacing [39]. Taken together, indiscriminate counting of actions of an ICD is no adequate surrogate, let alone intermediate parameter for SCD [40]. 

## 6. Large Intervention Studies

As yet, no randomized controlled trial on EPA+DHA has been published using SCD as a primary endpoint. However, the design of the OMEGA-trial has been published, and results were presented orally at the ACC meeting, spring 2009 [41]. The study size estimate of OMEGA was based on the assumption that SCD would occur in the first year in 3.5% of study participants in the placebo group, and in 1.9% of study participants in the intervention group that received 850 mg EPA+DHA/day in the form of an ethyl-ester (alpha: 2.5%, power 80%) [41]. Comparable to GISSI-P, patients were recruited shortly after a first myocardial infarction [42]. In the first year of GISSI-P, SCD occurred in 1.4% of the control group, which was reduced by 45% to 0.8% by 850 mg of EPA+DHA/ day in the form of an ethyl-ester [43]. These data of GISSI-P were known at the time when OMEGA was planned. It remains unclear why an inflated incidence of SCD was used for planning OMEGA. Not surprisingly, the actual incidence of SCD in OMEGA was 1.5% in the first year, and an effect of EPA+DHA could not be discerned (ACC Spring 2009). Because of the error in planning the study size, the actual power of OMEGA was 44%, and therefore was inadequate to base conclusions on. Nevertheless, the authors of OMEGA concluded that an effect of EPA+DHA could not be detected because of improvements in treatment of myocardial infarction since GISSI-P – a conclusion that cannot be reconciled with the actual data.

In GISSI-P, however, the primary endpoint was a combination of adverse cardiac events (death, non-fatal myocardial infarction, and stroke) [42]. GISSI-P was a 3.5 year, randomized, open label study in 11324 patients recruited shortly after a first myocardial infarction, comparing the additional effect of 850 mg EPA+DHA / day as an ethylester to a control group that received contemporary treatment after a myocardial infarction [42]. Overall, a 10% or 15% reduction in the primary endpoint was seen (p = 0.048 or p = 0.008, respectively, depending on the analysis (two way or four-way)). Upon further analysis, the most important contributor to the reduction in events was a reduction in SCD by 45% [42]. Time course analyses demonstrated this reduction to occur early, and to be significant already after 120 days [43]. GISSI-P was criticized for not having been conducted in a double-blind manner. However, conventional treatment post-myocardial infarction was evenly distributed between the groups. Therefore, it is hard to imagine that the absence of blinding has an impact on the occurrence of SCD. 

The first large randomized intervention study in patients with coronary artery disease was DART (Diet And Reinfarction Trial), which compared the advice to eat fatty fish twice weekly to other dietary advices in 2033 participants for two years [44]. Circumstantial evidence indicates that this increased weekly intake of EPA+DHA by some 6-7 g, similar to the other studies mentioned. After two years, overall mortality was reduced by 29%, mostly due to a reduction in fatal myocardial infarction [44]. The authors tried to replicate the results in men with angina, but, mostly due to problems in conducting the study, no interpretable results were obtained [45]. Thus, the DART trial supports the notion that overall, mortality is reduced by omega-3 fatty acids post myocardial infarction. 

The largest randomized intervention study to date, was JELIS, a five-year study comparing 1.8 g EPA ethylester (9326 participants) to no intervention (9319 participants) in hyperlipidemic Japanese [46]. The combined primary endpoint (SCD, fatal and non-fatal myocardial infarction, and other non-fatal events including unstable angina pectoris, angioplasty, stenting, or coronary artery bypass grafting) was reduced by 19 rel.% (p = 0.011). In both groups, the incidence of SCD was very low (40.8/100,000 participant years, lower than in a general Western population), and not reduced by the study intervention [46]. This is most likely due to the high levels of EPA+DHA that can be found in red cells from Japanese living in Japan [17]. Therefore, the results of JELIS rather argue for a protective effect of high levels of EPA+DHA in red cells than against it. 

More recently, GISSI-HF recruited patients with chronic heart failure of New York Heart Association class II–IV, and randomly assigned them to 850 mg EPA+DHA as ethylester daily (n = 3494) or to a matching placebo (n = 3481) [47]. Tolerability and safety of EPA+DHA and placebo were identical. After 3.9 years, the primary endpoints (time to death, and time to death or admission to hospital for cardiovascular reasons) were reduced by 9% (adjusted hazard ratio [HR] 0·91, 95·5% CI 0·833–0·998, p = 0·041), or 8%, respectively (adjusted HR 0·92, 99% CI 0·849–0·999, p = 0·009). Adjustment was predefined and due to baseline inequalities. In the intervention group, 274 participants (7·8%) died a presumably arrhythmic death, which was true for 304 participants (8·7%) in the control group (adjusted HR 0·88, 95% CI 0·75–1·04, p = 0.141). Sudden cardiac death occurred in 307 (9%) patients allocated to n-3 PUFA and 325 (9%) in the placebo group (adjusted HR 0·93 [95% CI 0·79–1·08], p = 0·333). While GISSI-HF illustrates the therapeutic value of EPA+DHA in congestive heart failure, it also demonstrates what an elusive endpoint SCD can be in clinical studies. 

SCD is a rare event, even in persons at high risk for cardiovascular events. Therefore, in order to detect a reduction in SCD, very large studies need to be conducted. Case estimates need to be performed on trial data, and not on registry data, since trial populations are known to have lower rates of events than registry populations. Inadequately powered trials are impossible to interpret. As discussed below, the use of the Omega-3 Index in trial design is likely to make more efficient studies possible. It is to be hoped that ongoing large and long trials, like ORIGIN [48] or ASCEND (both in diabetics), will be of sufficient size and duration to accrue a number of events to make more definite conclusions possible. However, for the only option to prevent SCD thus far established, *i.e,.* implantation of an ICD, only a minute fraction of cardiovascular patients qualify. This makes other preventive options a necessity. The trial data, especially if seen in conjunction with the epidemiologic data discussed above, and the well-documented safety and tolerability of EPA+DHA, indicate 850 mg EPA+DHA / day as a viable option towards prevention of SCD. As discussed below, the Omega-3 Index might provide a more focused approach towards prevention of SCD.

## 7. Meta-Analyses and Systematic Reviews

Reductions in SCD by EPA+DHA were reported between 19% [n.s., 49]  and 50% [50]. These figures are supported by reductions in total mortality between 14-19%, depending on the approach and timepoint of the meta-analysis or systematic review [22]. Most of the large intervention trials mentioned above were conducted with 850 mg EPA+DHA/day as an ethylester. When factoring in results of epidemiologic studies, some authors come to the conclusion that a daily dose of 250 mg EPA+DHA/per day is effective, while others consider doses higher than 500 mg/day to be necessary [50,51]. 

## 8. Current Guidelines

For prevention of SCD, EPA+DHA “may be considered” (level of recommendation IIb, class of evidence B), as stated in the joint guidelines of the European Society for Cardiology and American Heart Association [1]. This recommendation was based on the three studies in carriers of an ICD mentioned [1]. Interestingly, neither endpoint studies nor meta-analyses on the effects of EPA+DHA on incidence of SCD mentioned above were considered [1]. Future editions of these guidelines will hopefully be based on a more comprehensive review of the literature. 

## 9. Impact of the Omega-3 Index on Study Design and Results

In collaboration with the Framingham group and a number of other similar groups, several epidemiologic studies currently seek to relate the Omega-3 Index at baseline to SCD and other adverse cardiac events in a subsequent observation period [Harris WS, Störk S, personal communications]. Previously, epidemiologic studies were largely based on food frequency questionnaires that have inherent methodological problems discussed in more detail elsewhere “[von Schacky C “Prevention of cardiovascular disease–what can we learn from epidemiology?” in „Omega-3 fatty acids and cardiovascular diseases“, von Schacky, ed., Unimed, Bremen, London, Boston, 2010, in press]. Therefore, a clearer picture is likely to emerge from epidemiologic studies based on the Omega-3 Index than from epidemiologic studies based on dietary assessment.

A similar case can be made for the use of the Omega-3 index in intervention studies: In all populations studied so far, the Omega-3 Index had a normal (Gaussian) distribution. Therefore, a normal distribution of the Omega-3 Index can safely be assumed in the populations recruited for the large intervention studies at baseline. A sizable fraction of the study populations therefore had high levels, which implies that a benefit of the study intervention could not be expected, and few, if any, events are likely to occur in the control group. Conversely, a portion of participants in intervention group had low levels at baseline. Although they are likely to benefit from the study intervention, some will not reach optimal levels. Therefore, in both portions of participants – with high and low Omega-3 Index – some study participants are not likely to fully contribute to the study results, and bias the result towards neutral. A bias towards neutral will also occur, if the response to a fixed dose between individuals is variable, which has already been demonstrated [52]. A normal distribution of baseline levels and variability in response to a fixed dose will result in an overlap between the intervention and the control groups in terms of the Omega-3 Index, a further source of bias towards a neutral result. Non-compliance is likely to occur in any intervention group. When studying compounds like EPA+DHA that can be obtained outside the study setting, non-compliance is a distinct possibility in the control group, resulting in yet another bias of the study result towards neutral. Therefore, the large intervention studies discussed here all had a substantial bias towards a neutral result by design, since a low Omega-3 Index was not a criterion for recruiting participants, a target value was not defined, and compliance was not assessed by measurement of the Omega-3 Index during the study. In the future, recruiting study participants based on a low Omega-3 Index defining a target value for the Omega-3 Index, and assessing compliance during the study by means of the Omega-3 Index is likely to make studies more efficient and less prone to a bias towards neutral. 

## 10. The Omega-3 Index in Clinical Routine?

Currently, the MADIT-2 criteria are the only established criteria for implantation of an ICD, the only established means of prevention of SCD [1]. The vast majority of SCDs occur in persons not identified to be at risk for it. A similar, less pronounced situation exists in conventional risk assessment for cardiovascular events in general. While conventional scoring systems can identify persons at high risk based on conventional risk factor scoring systems [53], the number of cardiovascular events is larger in the persons with intermediate risk. Therefore, currently, a number of biomarkers are investigated and evaluated in order to better predict cardiovascular events. Among them are C-reactive Protein, coronary calcium, lipoprotein (a), homocysteine, and many others. The US Preventive Service Task Force and the American Hearth Association both have issued sets of criteria to be fulfilled by novel biomarkers [54,55]. 

Among these criteria, standardization and ease of measurement, availability, cost, and safety were thought to be important. Clearly, laboratory parameters have advantages here, because they are standardizable, and can be made widely available to practitioners by shipment of blood samples. In contrast, interrogator-dependent assessments, like intima-media thickness, coronary calcium scoring, or ankle-brachial index, are less well standardizable, and availability can be a problem. Safety, may be a concern for coronary calcium scoring, because of radiation exposure. Standardization of the Omega-3 Index is discussed above. Therefore, the criteria mentioned here are met by the Omega-3 Index.

Two criteria, *i.e.,* “incremental informative value” and “reclassification” were thought to be especially important [54,55]. Both criteria call for epidemiologic studies with assessment of the novel biomarker at baseline, and follow-up on clinical events during the observation period.

Incremental information is obtained, if the information provided by the novel biomarker adds information to the risk classification provided by conventional risk factor based risk assessment, like Framingham or the ESC-Score [53]. This can be evaluated by the c-statistic, where incremental information can be quantified as an increase in “area under the curve” in comparison to the area under the curve provided by a conventional risk assessment. A first assessment of the c-statistic in patients with acute coronary syndrome provided evidence that information contained in the fatty acid spectrum of red blood cells, as analyzed with the Omega-3 Index methodology, increased the area under the curve [56]. 

Reclassification describes the identification of a person, labelled “intermediate risk” by conventional risk assessment as “high risk”, based on the information of the novel biomarker. Information contained in the fatty acid spectrum of red blood cells, as analyzed with the Omega-3 Index methodology, increased reclassification rates [56]. 

A case-control study on the association between the Omega-3 Index and subsequent SCD is ongoing in collaboration with ORIGIN, a large intervention study with omega-3 fatty acids in persons with diabetes [48]. This and other work in progress in collaboration with the Framingham group and other groups will determine the value of the Omega-3 Index in increasing informative value (the area under the curve) and reclassification rates. Evidence obtained so far indicates that a high Omega-3 Index (>8%) is associated with a low risk for cardiovascular events, including SCD, and a long life expectancy [7,21,57].

Moreover, determination of the novel biomarker should have a therapeutic consequence with proven value – a criterion partially fulfilled by determination of C-reactive Protein, but by none of the other novel biomarkers [54,55,58]. However, as discussed above, increased consumption of EPA+DHA, resulting in an untargeted increase of the Omega-3 Index, has already been demonstrated to prolong life and to reduce major adverse cardiac events, like SCD. Whether an approach targeting an Omega-3 Index >8% in order to reduce SCD or other cardiovascular events is superior to an untargeted increase, remains to be evaluated, however. 

## 11. Conclusion

Taken together, SCD is a major unabated source of mortality, mainly because assessing risk for SCD has remained an elusive goal. The Omega-3 Index is a good candidate for a biomarker to assess risk for SCD and other major adverse cardiac events. Important criteria like standardization, availability, and safety are fulfilled by the Omega-3 Index. Work in progress will determine incremental informative value and reclassification rates of the Omega-3 Index. Evidence from large intervention trials indicates that SCD can be prevented by increased intake of omega-3 fatty acids. The use of the Omega-3 Index provides a more targeted approach in epidemiologic and intervention studies, yet to be fully exploited. 
